# Imaging foreign bodies in head and neck trauma: a pictorial review

**DOI:** 10.1186/s13244-021-00969-9

**Published:** 2021-02-15

**Authors:** Jan Oliver Voss, Christoph Maier, Jonas Wüster, Benedicta Beck-Broichsitter, Tobias Ebker, Jana Vater, Steffen Dommerich, Jan D. Raguse, Georg Böning, Nadine Thieme

**Affiliations:** 1 Department of Oral and Maxillofacial Surgery, Charité – Universitätsmedizin Berlin, corporate member of Freie Universität Berlin, Humboldt-Universität zu Berlin, and Berlin Institute of Health, Augustenburger Platz 1, Berlin, 13353 Germany; 2 Berlin Institute of Health (BIH), Anna-Louisa-Karsch-Straße 2, 10178 Berlin, Germany; 3 Department of Radiology, Charité – Universitätsmedizin Berlin, corporate member of Freie Universität Berlin, Humboldt-Universität zu Berlin, and Berlin Institute of Health, Augustenburger Platz 1, Berlin, 13353 Germany; 4 Department of Otorhinolaryngology, Charité – Universitätsmedizin Berlin, corporate member of Freie Universität Berlin, Humboldt-Universität zu Berlin, and Berlin Institute of Health, Charitéplatz 1, Berlin, 13353 Germany; 5 Department of Oral and Maxillofacial Surgery, Fachklinik Hornheide, Dorbaumstraße 300, 48147 Münster, Germany

**Keywords:** Foreign body injuries, Imaging foreign bodies, Penetrating injury, Dislocated foreign body

## Abstract

Open injuries bear the risk of foreign body contamination. Commonly encountered materials include gravel debris, glass fragments, wooden splinters or metal particles. While foreign body incorporation is obvious in some injury patterns, other injuries may not display hints of being contaminated with foreign body materials. Foreign objects that have not been detected and removed bear the risk of leading to severe wound infections and chronic wound healing disorders. Besides these severe health issues, medicolegal consequences should be considered. While an accurate clinical examination is the first step for the detection of foreign body materials, choosing the appropriate radiological imaging is decisive for the detection or non-detection of the foreign material. Especially in cases of impaired wound healing over time, the existence of an undetected foreign object needs to be considered.

Here, we would like to give a practical radiological guide for the assessment of foreign objects in head and neck injuries by a special selection of patients with different injury patterns and various foreign body materials with regard to the present literature.

## Key points

1. Any unnoticed foreign body harbours the risk of infection or other complications.
2. Glass, metal and stone are radiopaque and readily visualised in X-ray/CT.
3. Wood is commonly hypodense in CT and easily missed in X-ray.
4. “Plastic“ encompasses a wide spectrum of materials and may be undetectable in CT.
5. Ultrasound and MRI are valuable tools if an object is occult on X-ray/CT.

## Background

The head and neck areas are complex anatomical regions, comprising vulnerable organ systems including vascular and nervous structures, the aero-digestive tract, as well as the auditory and visual systems. Like the hands, the head and neck and the face in particular are usually exposed to the external environment. Therefore, injuries to these regions are common and carry a higher risk of foreign body contamination, compared with other (usually clothed) body parts. Wood, metal and glass are the most commonly incorporated materials [1]. Foreign objects can penetrate the skin or the eye globe and can also enter the head and neck through the mouth, nostrils and external auditory meatus. They may reach into the orbits, paranasal sinuses or the deep spaces of the head and neck. A wide variety of accidental, self-inflicted, iatrogenic or assault-related incidents involving foreign bodies have been reported in the literature [2–5]. The range of trauma mechanisms includes direct penetration, inhalation and swallowing [6–10].

Foreign body injuries carry a risk of acute and potentially life-threatening complications such as bleeding, airway compromise or neurovascular injury [11–14]. While some retained foreign bodies may remain clinically silent for years or even indefinitely, there is a general risk of late sequelae, primarily in the form of infections, which can result in persisting impairment [7, 15–17]. Delayed complications include persisting pain, impaired wound healing and inflammatory responses with potential abscess formation, fistulas, necrotising fasciitis and foreign object migration [4, 18–23].

Besides adverse clinical outcomes, undetected foreign bodies may also result in medicolegal consequences if the patient takes legal action [24, 25].

Therefore, it is mandatory to detect any foreign body during clinical workup. In many cases, the detection of foreign bodies is not a clinical challenge, e.g. in impalement injuries, small gravel debris in superficial wounds or penetrating injuries. In other scenarios, however, the foreign body incorporation may not be obvious at first sight. Foreign bodies lodged in deep wounds or in body orifices may present with minimal or no clinical complaints, falsely leading the physician to disregard the possibility of a foreign body injury. For hand injuries, Anderson et al. reported that 38% of soft tissue foreign bodies were not detected at the time of initial presentation [15]. Therefore, a high index of suspicion is necessary to ensure proper management.

A thorough investigation of the patient’s medical history and clinical examination are the first and crucial steps in the workup. It is important to collect information on the trauma mechanism and, if applicable, the exact nature of involved objects. Even the conditions of a surface material may be relevant in the case of falls and traffic accidents [26].

A preliminary diagnosis gained from the history and clinical examination facilitates the decision if imaging studies are warranted and guides the choice of optimal modality. However, regarding the high incidence of foreign body injuries in paediatric patients, an insufficient patient history and/or clinical examination might lead to a missed foreign object. In paediatric patients, foreign bodies are typically ingested, inhaled or manually inserted, depending on the age group [27].

Imaging studies can significantly increase the chance of detecting a foreign body [1]. They can also aid in surgical planning by precisely determining the anatomical location of the foreign object and its spatial relation to neighbouring structures, thereby reducing the risk of collateral damage [28].

Plain radiographs, computed tomography (CT), ultrasound and magnetic resonance imaging (MRI) can be used for the detection of foreign objects. Choosing the ideal modality primarily depends on the chemical composition of the suspected foreign object and on its presumed anatomical location. Successful detection is also aided by skillful radiographers and by radiologists who are knowledgeable in imaging the features of foreign bodies.

In this review, we will discuss the imaging of suspected foreign bodies in the head and neck areas. Particular attention will be paid to special characteristics of different imaging modalities (Section I) and the imaging characteristics of materials that are commonly encountered in clinical practice (Section II). A selection of patient cases from our institution will highlight important clinical and radiological features of foreign body injuries.

## Section I—imaging modalities

Each imaging modality has strengths and limitations, and the wide spectrum of physico-chemical properties of various materials outlined in Section II implies that there is not one single ideal imaging modality. The most appropriate imaging study must be chosen based on the suspected material and its anatomic location. Moreover, radiation exposure, cost, availability and patient-specific limitations (e.g. inability to cooperate or the presence of ferromagnetic implants) need to be considered. MRI in particular may not be available 24/7 in an emergency setting.

Conventional X-ray imaging is probably the most frequently used method for the detection of radiopaque foreign body materials [1]. This modality is fast and readily available in most hospitals. However, ultrasound, CT and MRI also have their unique advantages. The strengths and limitations of each modality will be outlined below.

Various studies have analysed the competencies of different imaging modalities using common foreign body materials in different experimental settings. However, differences in study design have led to discrepant results regarding what the best imaging modality is for specific types of foreign body materials [29–36].

Even if a foreign body cannot be visualised directly on a given modality due to its chemical composition, there may be image artefacts that hint at its presence. Sequelae like emphysema, haematoma, foreign body granuloma, inflammatory reactions or even frank abscesses can provide further (albeit non-specific) clues suggesting the possibility of a foreign body injury.

### Conventional X-ray

Conventional X-ray imaging is widely used and accessible in virtually every emergency room, providing an excellent and fast overview of an anatomic region. Dense materials like metal, glass or stone are clearly depicted on X-ray [29, 37]. Foreign bodies composed of wood or plastic on the other hand are challenging to visualise by X-ray because their density is similar to that of soft tissue. Of note, the detectability also depends on the surrounding tissue (soft tissue vs air vs bone) [29, 31, 32]. In Case No. 1, for example, the dislocated wooden stick was not visible in conventional X-ray, which resulted in a delayed detection.

### CT

CT scanning is considered the gold standard in foreign body imaging [38]. Compared with conventional X-ray techniques, cross-sectional CT images improve detectability and allow for precise anatomic localisation of foreign body materials. Furthermore, a three-dimensional CT dataset can be used with intraoperative navigation systems in order to facilitate surgical removal [39]. CT is readily available and can also rule out fractures or other concomitant injuries in a single examination, which is particularly valuable in polytraumatised patients.

CT can easily detect radiopaque objects like metal, stone and glass, and it may also help visualise radiolucent objects such as plastics, wood or other organic materials that are invisible to X-ray imaging due to summation effects (Case No. 1). Depending on the surrounding tissue however, radiolucent materials can be virtually invisible, even in CT.

Hence, it is important to watch out for indirect signs mentioned above. Contrast enhanced CT can accentuate these indirect signs and provide further information on vascular injuries or active bleeding. This aids in estimating injury severity and in surgical planning. It is also worth noting that cone beam CT/digital volume tomography systems, which are commonly used by dentists and maxillofacial surgeons, suffer from inferior soft tissue contrast. Thus, a conventional (fan beam) CT is preferable for the detection of non-radiopaque objects.

In patients carrying metallic implants other than the presumed foreign body (most commonly dental hardware), metal-related artefacts can significantly impair the detection of foreign bodies. While an artefact reduction software can improve image quality, the results must be interpreted with caution because the algorithms might erroneously “remove” metallic objects [40]. In the context of a suspected foreign body, it is therefore essential to evaluate source images as well as post-processed images. The advantages of CT over X-ray imaging should be weighed against their significantly higher radiation doses, depending on the clinical scenario.

### Ultrasound

Like conventional X-ray imaging, ultrasound scanning is available in most emergency departments. When being performed at the point-of-care during wound care or intraoperatively, ultrasound has the unique advantage of providing immediate confirmation of complete foreign body removal [41]. Foreign objects are generally hyperechoic; reverberation artefacts may therefore provide further clues about their presence and can be enhanced with Doppler imaging [42].

Ultrasound has been shown to reliably detect wood and plastics, where X-ray and CT imaging may fail [32, 43]. Ultrasound scanning is well suited for assessing superficial tissues where it can afford even higher spatial resolutions than CT imaging or MRI [29]. The evaluation of deeper structures, on the other hand, may be impossible if the region of interest is obscured by bone or air. The field of view is also limited by the penetration depth of the acoustic waves.

The diagnostic performance of ultrasound generally depends on the skill of the examiner, and it is considered to be less reproducible than other modalities. While routine enquiries regarding foreign bodies can be handled by most examiners, specific questions about foreign bodies in the head and neck may exceed the expertise of emergency medicine physicians who are not specialised in maxillofacial trauma.

### MRI

In clinical practice, MRI is only infrequently used to detect or exclude the presence of foreign bodies, mainly due to cost and availability barriers. Moreover, safety issues must be considered given that ferromagnetic objects are subject to torque and translation forces in the static magnetic field and can also undergo radiofrequency-induced heating. Indeed, inadvertent exposure of metallic foreign objects to the MRI environment can result in adverse events, potentially injuring neighbouring structures [44]. Since ferromagnetic materials (primarily iron and steel alloys) are invariably radiopaque, it is advisable to perform X-ray or CT imaging prior to MRI to rule out the occurrence of metallic foreign bodies, especially in critical locations such as the orbits [45]. While not all metals are ferromagnetic, the exact material composition of a foreign object cannot usually be determined reliably in a trauma setting. Therefore, metallic foreign objects should be considered ferromagnetic unless proven otherwise.

Despite these drawbacks, MRI can be a valuable tool for detecting foreign bodies. In the case of objects which are radiolucent and also lodged in anatomical locations which are inaccessible to ultrasound scanning, MRI may even be the only modality capable of enabling their successful detection [46].

Even materials which are safe to undergo MRI examination can result in image artefacts secondary to magnetic susceptibility effects [47]. In general, susceptibility artefacts are undesirable because they degrade image quality. When seeking a foreign body, however, susceptibility artefacts should be deliberately searched for since they may be more conspicuous than the foreign body itself. The magnitude of susceptibility artefacts depends on the sequence type (gradient echo, echo-planar imaging and susceptibility weighted imaging are more affected than spin echo sequences), and it increases with field strength and echo time (Port et al., 2000). Lower receiver bandwidths also result in greater susceptibility effects. Taking these parameters into account, imaging protocols for suspected foreign bodies can be tailored to include susceptibility-prone sequences.

## Section II—Foreign body materials

### Wood

Wood is an organic material composed of layers of fibres with a porous structure. The imaging appearance of wood can be influenced by the porosity of the wood and by its relative gas/water content. Dry wood has a lower water content and therefore lower density than fresh (green) wood or wood fragments which have been embedded intracorporally for a significant time [48]. In a dry state, only very few hard woods such as lignum vitae or ebony can reach a density exceeding 1 g/cm^3^ (equivalent to 0 Hounsfield units on CT) [49]. Industrial processing of wood products can also further alter their imaging appearance (Case No. 2).

The detectability of wooden foreign bodies can be also be affected by their anatomical location [33, 48]. Even within the same imaging modality (CT and MRI), imaging characteristics will differ depending on the water/gas ratio of the compound. In a study by Pattamapaspong et al., the success rate in detecting fresh wooden foreign objects was higher in CT scans compared with dry wood objects which were more likely detected in MRI scans [35]. Mizel and colleagues analysed wooden splinters of various sizes soaked in saline water for a duration of 3 days or 5 months in an experimental setting used to mimic acute injury (dry) or chronically embedded (wet) wooden foreign objects in muscle tissue. Overall, ultrasonography and MRI were shown to be superior to X-ray and CT imaging independent of the soaking time [33].

In contrast, Ingraham et al. analysed different wooden objects in both dry and wet set-ups and concluded that none of the wooden objects, regardless of dry/wet conditions, were visible in MRI scans [31]. Similarly, Javadrashid et al. were unable to visualise pieces of dry wood of smaller than 3 mm using MRI [32].

In ultrasound scanning, wood can be detected reliably as it is usually hyperechoic [31, 50]. In superficial wounds, ultrasound scans offer a favourable alternative to plain radiographs [15, 51] because wooden foreign objects are at high risk of being overlooked in conventional X-ray imaging [1, 29, 48, 50].

Missed wooden foreign objects can lead to severe bacterial infections due to their mixed bacterial flora contents, including *Escherichia coli*, *Escherichia vulneris*, *Bacillus cereus*, gamma-haemolytic *Streptococci* and *Enterococcus durans* as well as *Clostridium perfringens*. Bacterial infections can be complicated by severe inflammatory reactions, abscess formations, emphysema due to gas-forming bacteria or even necrotising fasciitis [23, 52]. These complications should be considered as possible imaging manifestations of retained foreign bodies in chronic wounds.

In Case No. 1, the wooden foreign object is hypodense and could be overlooked in a cursory image review. However, surrounding phlegmon/abscesses and emphysema increase its conspicuity.

### Case No. 1: Wooden stick

**Fig. 1:**
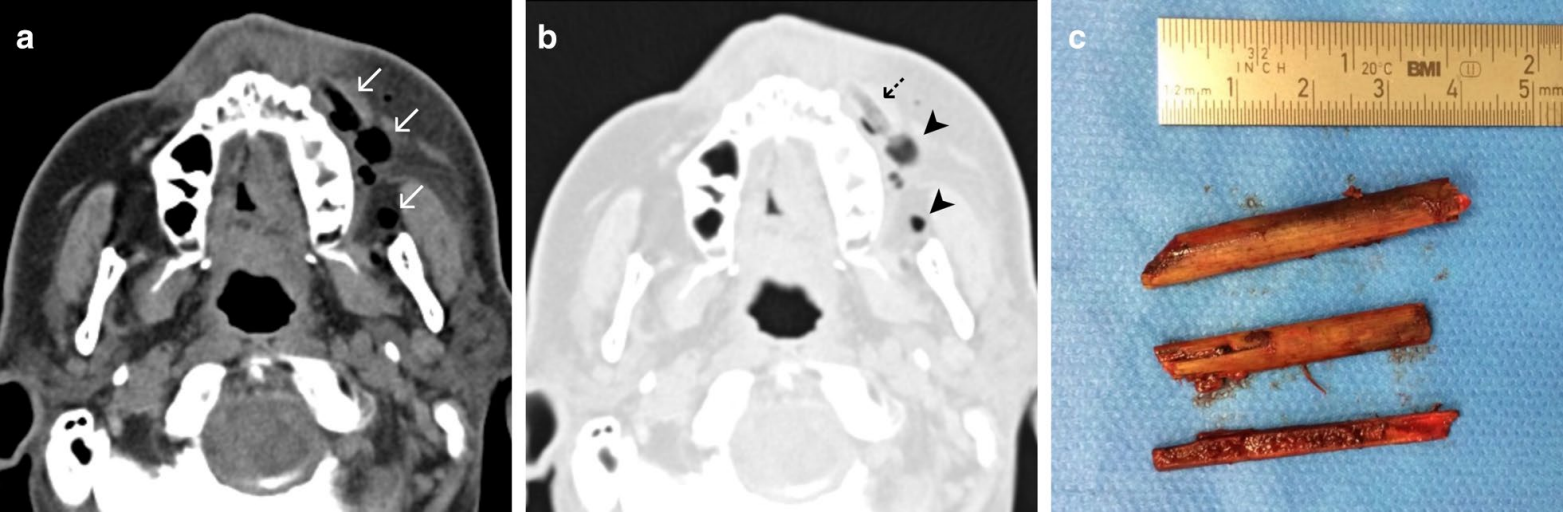
Wooden stick. Axial CT image in soft tissue window (**a**) shows phlegmonous fat stranding in the left buccal region, surrounding multiple hypodense features (arrows), giving the impression of emphysema. Closer inspection of the lung windows (**b**) reveals that one of the hypodense structures has a discernible internal structure (dashed arrow), distinguishing it from the homogenously hypodense gas locules (arrowheads). Three pieces of a wooden stick were surgically removed (**c**)

A 60-year-old male patient presented to our emergency department with a persisting swelling of his left cheek after an injury to his face 6 days earlier. An initial workup at a different hospital included a physical examination and CT scan, but no treatment was initiated. The patient presented with purulent discharge from his left nostril and a tender cheek. A detailed clinical history revealed that he had fallen into a vegetable patch. Conventional X-ray imaging showed no signs of a foreign body. Considering the clinical picture, the external CT scan was re-evaluated, and special attention was paid to the possibility of a foreign body that might have been overlooked during the initial workup. CT imaging demonstrated the presence of multiple hypodense structures and surrounding phlegmon in the left buccal region (Fig. 1a, b). Closer inspection revealed that some of the hypodense structures represented the wooden objects, whereas others represented emphysema. Multiple pieces of a wooden stick were removed under general anaesthesia (Fig. 1c).

### Case No. 2: Chipboard wood

**Fig. 2:**
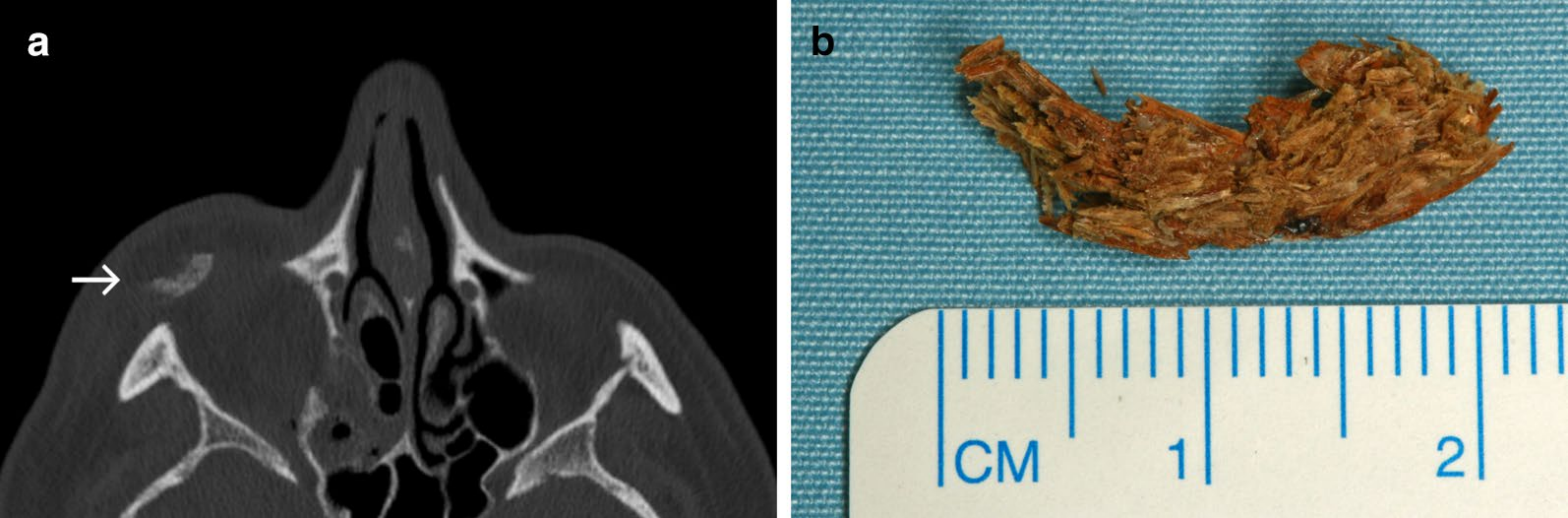
Chipboard wood. **a** An axial CT image displaying a hyperdense structure dislocated in periorbital soft tissues inferior and anterior to the right globe (arrow). Hyperdensity of the foreign object in this case is likely due to its industrial processing. **b** A photograph of a chipboard fragment after its removal from the soft tissues

An 80-year-old patient presented to our emergency department after falling into a cupboard, injuring his lower eyelid. Palpation and blunt examination with forceps revealed a foreign body below skin surface of the lower eyelid. CT imaging demonstrated a blow-out fracture of the right orbital floor and a periorbital hyperdense foreign object inferior to the right globe (Fig. 2a). A 2 × 1-cm-long wooden chipboard piece was removed under local anaesthesia in the emergency room (Fig. 2b). Orbital floor reconstruction was achieved under general anaesthesia. Further ophthalmological evaluation showed no further injury to the eye. The patient was discharged after surgery without further complications.

### Case No. 3: Intraorbital wood

**Fig. 3:**
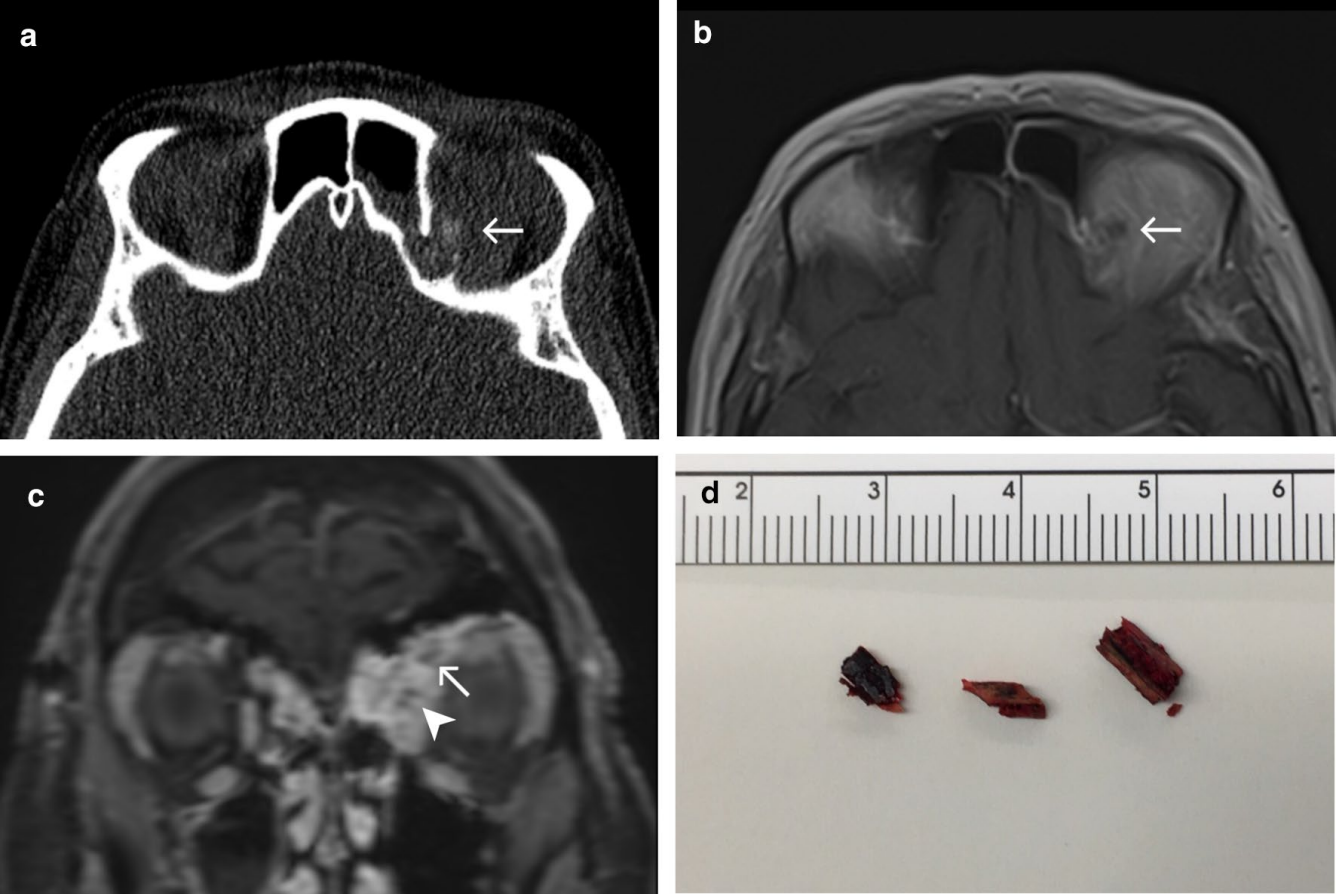
Intraorbital wood. Axial CT image (**a**) and axial T1-weighted MRI post-contrast (**b**) demonstrate a small structure close to the orbital roof (arrow), which is moderately hyperdense and hypointense. There is a bony defect in the adjacent wall of the frontal sinus. Coronal T1-weighted MRI with fat saturation post-contrast (**c**) reveals the aforementioned object (arrow), two more small punctate hypointense foci (arrowheads) and extensive enhancement of the surrounding orbital soft tissues and sinus mucosa. Differential diagnosis for the hypointense structures at this point includes the presence of foreign objects and dislocated bone fragments. Based on MRI alone, abscesses and emphysema should also be considered, but the appearance of a hyperdense object in CT images helps narrow the differential in this case. **d** A photograph of three wood pieces after their surgical removal

A 55-year-old female was referred to our hospital with delayed recovery after an impalement injury to the left orbit three months earlier. Initial treatment had been performed at a different facility, but the patient was left suffering from persisting diplopia and ptosis. CT and MRI scans were obtained and multiple hypointense/hyperdense structures hinted at the possibility of retained foreign objects (Fig. 3a–c). Surgical exploration revealed a chronic inflammatory reaction and yielded the recovery of three pieces of wood (Fig. 3d). The patient’s condition improved with further post-operative antibiotics.

#### Tooth fragments and dental prosthetics

Tooth fragments, metal and ceramic crowns and conventional dentures might be best visualised using conventional X-ray imaging, cone beam CT or CT scans due to their radiopaque appearance [32, 53]. Considering the radiation dose however, ultrasound scans might offer a safer alternative, especially for imaging soft tissue wounds occurring in regions like lips or cheeks [32, 54]. MRI scans of patients with broken orthodontic devices including brackets, wires or loose removable dentures should only be performed after considering their ferromagnetic characteristic because the MRI safety of these objects can be impaired if they are not attached securely [55, 56]. In Case No. 4, hyperdense objects are visible within soft tissue. There is a clear difference in signal between prosthetic restorations and teeth.

### Case No. 4: Broken teeth

**Fig. 4:**
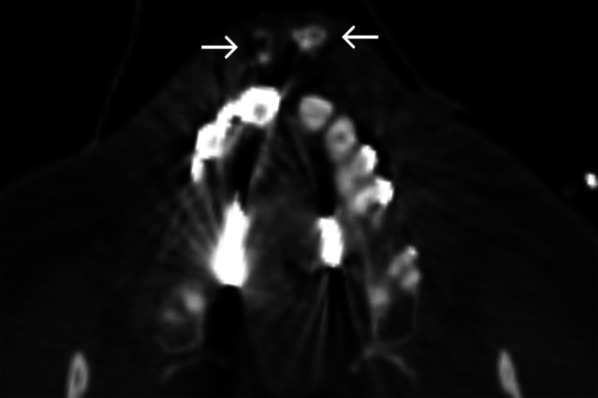
Broken teeth. Axial CT image showing multiple dislocated hyperdense objects in the upper lip (arrows)

A 66-year-old male patient was transferred to the emergency room after a motorcycle accident. Clinically, the upper lip was injured with a 3-cm-long cut. Intraoral examination revealed fractured medial incisors of his maxilla. Considering the clinical picture, special attention was paid during imaging to the potential presence of dislocated dental foreign objects. Here, a polytrauma CT scan enabled the detection of multiple hyperdense foreign objects (Fig. 4). Dental crown fragments were removed under local anaesthesia.

#### Metal

The spectrum of metal foreign body injuries includes a broad range of traumatic larger objects e.g. bullets, metals parts from improvised explosive devices, blades, drilling devices or keys [14], but smaller objects such as wires, metallic splinters, incorporated batteries or coins are also regularly seen in our hospital, requiring a diligent clinical and morphological imaging procedure. Overall, metal foreign bodies are more or less easy to detect depending on their overall size and anatomical location [32].

Ultrasound, conventional X-ray and CT imaging can readily help in visualising metallic foreign objects with a comparable detection rate in experimental settings [29, 57]. However, ultrasound scanning has its limitations in detecting smaller objects compared to conventional X-ray and CT scans [32]. In MRI, ferromagnetic objects are subjected to safety considerations outlined above. Upon imaging, metal foreign bodies generally exhibit signal loss and various degrees of susceptibility artefacts, depending on the size and chemical composition of the object and on the pulse sequence [58].

In Cases No. 5–9, various dislocated metal foreign bodies are displayed, including a carpet nail, paperclip, splinter of a broken hammer, nail and wire.

### Case No. 5: Carpet nail

**Fig. 5:**
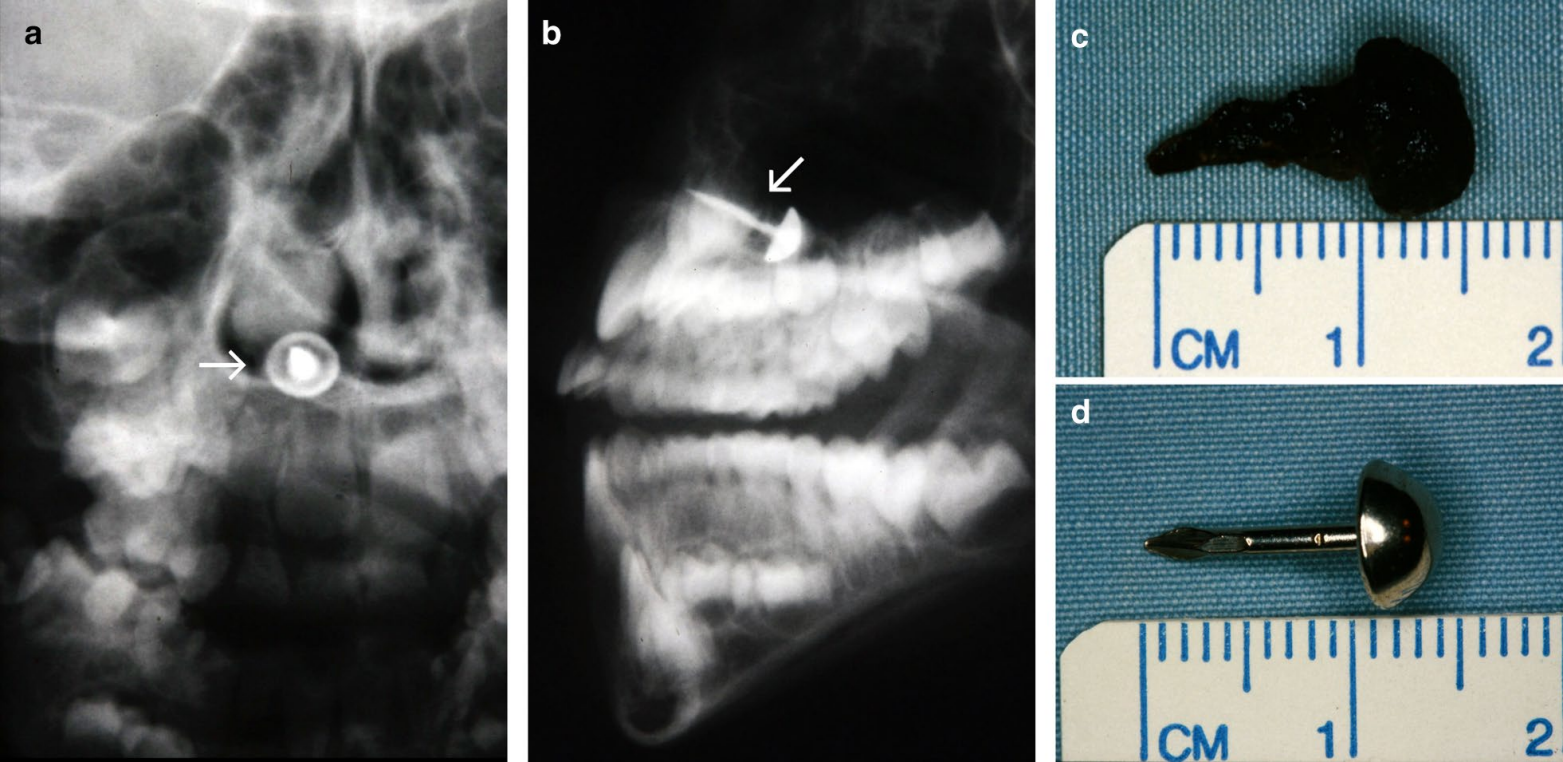
Carpet nail. Anteroposterior (**a**) and lateral (**b**) radiographs displaying a radiopaque object (arrow) in projection over the lower right nasal meatus. Photographs of a dislocated carpet nail after removal from the lower meatus (**c**, **d**). Adherent blood clots can impair the clinical detection of foreign objects

A 6-year-old boy was admitted to our emergency department after falling down the stairs, sustaining a monocle haematoma in his right eye. Conventional X-ray imaging was performed to rule out bone injuries. Unexpectedly, the X-ray scan detected a dislocated radiopaque foreign body in projection over the right lower nasal meatus (Fig. 5a, b). The foreign body was removed under general anaesthesia (Fig. 5c, d). Upon further questioning, the parents reported a fall on a carpet a month prior, but they had not noticed any missing carpet nails. The patient was discharged without any complications.

### Case No. 6: Paper clip

**Fig. 6:**
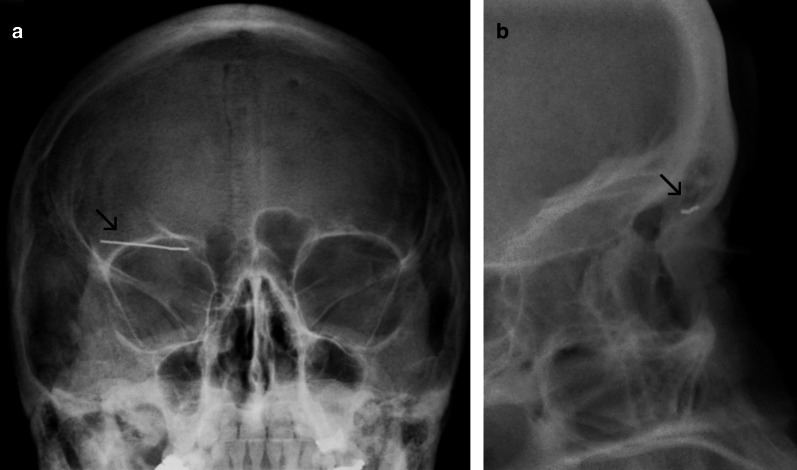
Paper clip. Anteroposterior (**a**) and lateral (**b**) radiographs showing a linear radiopaque structure in projection of the upper eyelid (arrow)

A 49-year-old female presented with a swelling of her right eyelid. The patient reported the insertion of a metal paper clip into her upper eyelid alongside other self-manipulating incidents in the past. Conventional X-ray confirmed the presence of a radiopaque foreign object in projection of the upper eyelid (Fig. 6). Foreign body removal was achieved under local anaesthesia.

### Case No. 7: Broken hammer

**Fig. 7:**
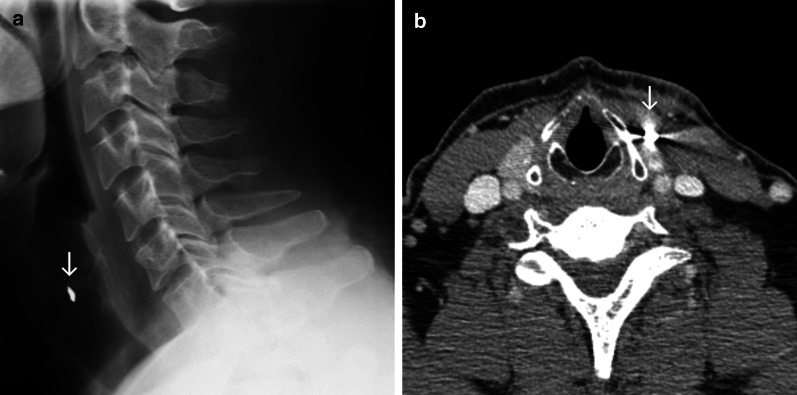
Broken hammer. Lateral radiograph (**a**) and axial contrast enhanced CT image (**b**) showing a radiopaque/hyperdense structure (arrow) anteromedial to the sternocleidomastoid muscle and lateral to the thyroid cartilage. Streak artefacts in the CT image indicate marked density, a potential clue that the foreign object is metallic

A 40-year-old male patient was admitted to the emergency department with an injury to his neck. The patient reported that a piece of a hammer had fractured and become dislocated into his neck, while he was working with metal nails. Clinically, the patient presented a 1-cm-long wound in the neck close to the larynx. Conventional X-ray scans were obtained and revealed a radiopaque structure in the neck (Fig. 7a). A CT scan was carried out for further preoperative planning. Here, a hyperdense structure was found in the infrahyoid musculature lateral caudal to the thyroid cartilage and in close proximity to the upper pole of the thyroid gland (Fig. 7b). Vascular injuries or active bleeding could not be detected. Under general anaesthesia, a 9 × 6 mm metal foreign body was removed.

### Case No. 8: Nail gun

**Fig. 8:**
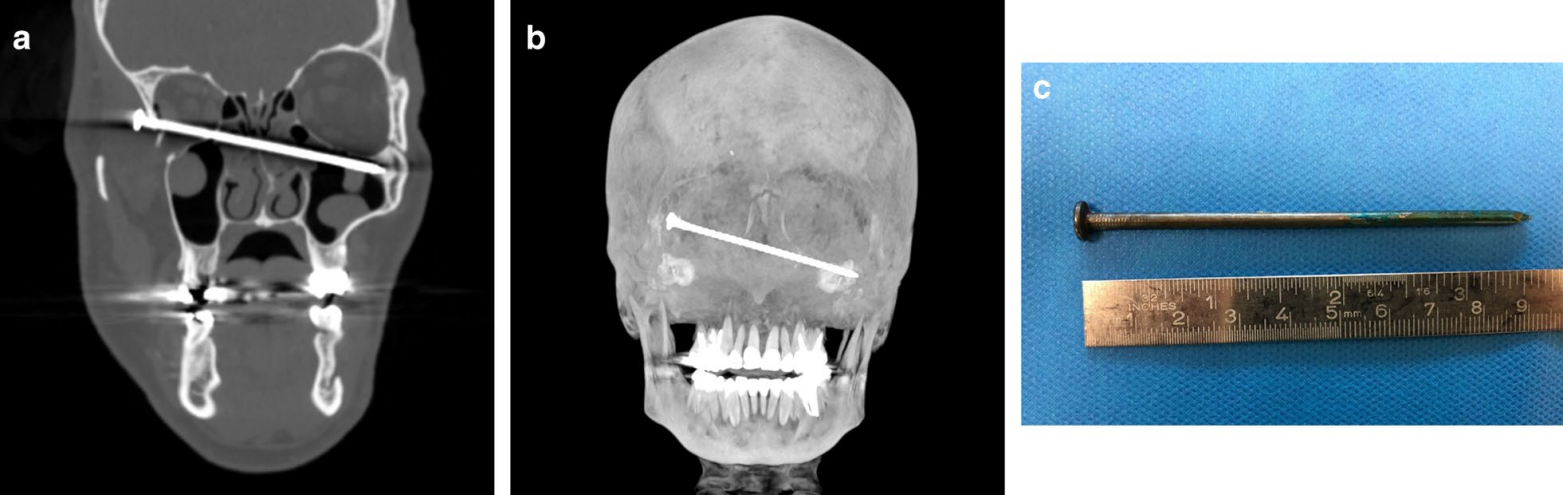
Nail gun. Para-coronal CT image (**a**) and a 3-dimensional maximum intensity projection (**b**) showing a hyperdense straight foreign object entering the right lateral wall of the orbit, penetrating the left ethmoidal cells and the left maxillary sinus. A photograph of a metal nail after removal from the midface (**c**)

A 56-year-old patient was admitted to the emergency room with a wound to his right temple. Third party anamnesis reported a suicide attempt by a self-inflicted nail gun injury. A contrast-enhanced CT scan presented a 9-cm-long hyperdense object in the right temporal region penetrating through the lateral orbital wall and entering the nasal cavity/ethmoidal cells and left maxillary sinus (Fig. 8a, b). There were only minimal intraorbital haematoma and emphysema on imaging. With the patient exhibiting stable vital signs, foreign body removal was achieved using general anaesthesia and a temporal and intraorbital approach (Fig. 8c). The post-operative outcome was satisfying without major complications and with preserved vision.

### Case No. 9: Metal wire

**Fig. 9:**
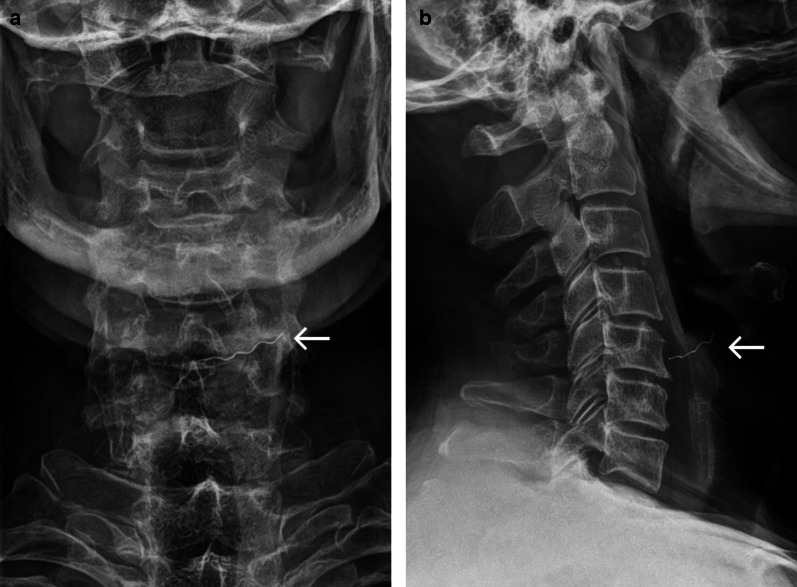
Metal wire. Anteroposterior (**a**) and lateral (**b**) radiographs showing a thin radiopaque structure (arrow) in projection over the hypopharynx anterior to the 5th cervical vertebrae

A 69-year-old patient was admitted to the emergency room with odynophagia and a foreign body sensation in his throat after eating a doner kebab hours earlier. Conventional X-ray imaging presented radiopaque foreign body material in projection anterior to the 5^th^ cervical vertebra (Fig. 9). Under general anaesthesia, an approximately 3-cm-long metal wire was removed from the left piriform recess. No further complaints were reported afterwards.

#### Glass

Glass is a family of chemical compounds that include silicate glass, aluminosilicate glasses or other oxide additives, glass–ceramics and fibre glasses. These different types of glass exhibit different physical characteristics which need to be considered in diagnostic imaging. There is a widespread misbelief that glass is not visible in conventional X-ray. This view has often resulted in reluctance to obtaining radiographic images and has led to malpractice claims as a consequence [25]. This misconception was mainly based on the assumption that glass needs to contain lead to be opaque on radiographs [59].

Today, it is recognised that glass foreign bodies are generally radiopaque and display good visibility in conventional X-ray imaging as well as in CT scans, with some variability based on size and surrounding tissues [29, 31, 32, 60]. In ultrasound scanning, alongside various artefacts, a hyperechoic signal with a strong interface can be detected [29, 34]. Data about the visibility of glass in MRI scans are inconsistent. Javadrashid and colleagues as well as Oikarinen and colleagues reported an insufficient detectability, while Ingraham and colleagues found glass to be detectable, secondary to artefacts [31, 32, 34].

Cases No. 10–12 present different dislocated glass foreign objects including a broken beer bottle, broken ophthalmic lenses or broken parts of a water glass. In these cases, imaging was mainly conducted using CT, and in one case, conventional X-ray was followed by CT scan.

### Case No. 10: Glass bottle

**Fig. 10:**
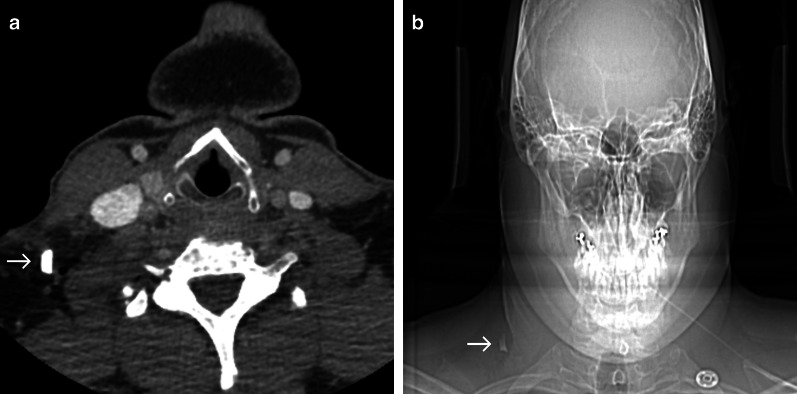
Glass bottle fragment. Axial contrast enhanced CT image (**a**) and corresponding anteroposterior scout scan (**b**) displaying a hyperdense structure (arrow) in the right posterior cervical space dorsolateral to the sternocleidomastoid muscle. At first glance, this object could look like a metal fragment (compare Fig. 7b). Careful comparison, however, reveals a lack of streak artefacts in CT images which would be expected in metallic objects of this size

A 34-year-old patient presented to the emergency department with a neck injury following a tumble fall sustained while leaving a train carrying a glass bottle. Clinically, multiple glass fragments could be removed superficially. A contrast enhanced CT scan was carried out to rule out injuries to the deeper cervical tissues. CT imaging presented a hyperdense object (13 × 8 mm) dorsolateral to the sternocleidomastoid muscle with air inclusions (Fig. 10). Under general anaesthesia, a glass fragment with aluminium foil attached to it was removed without further complications.

### Case No. 11: Broken ophthalmic lens

**Fig. 11:**
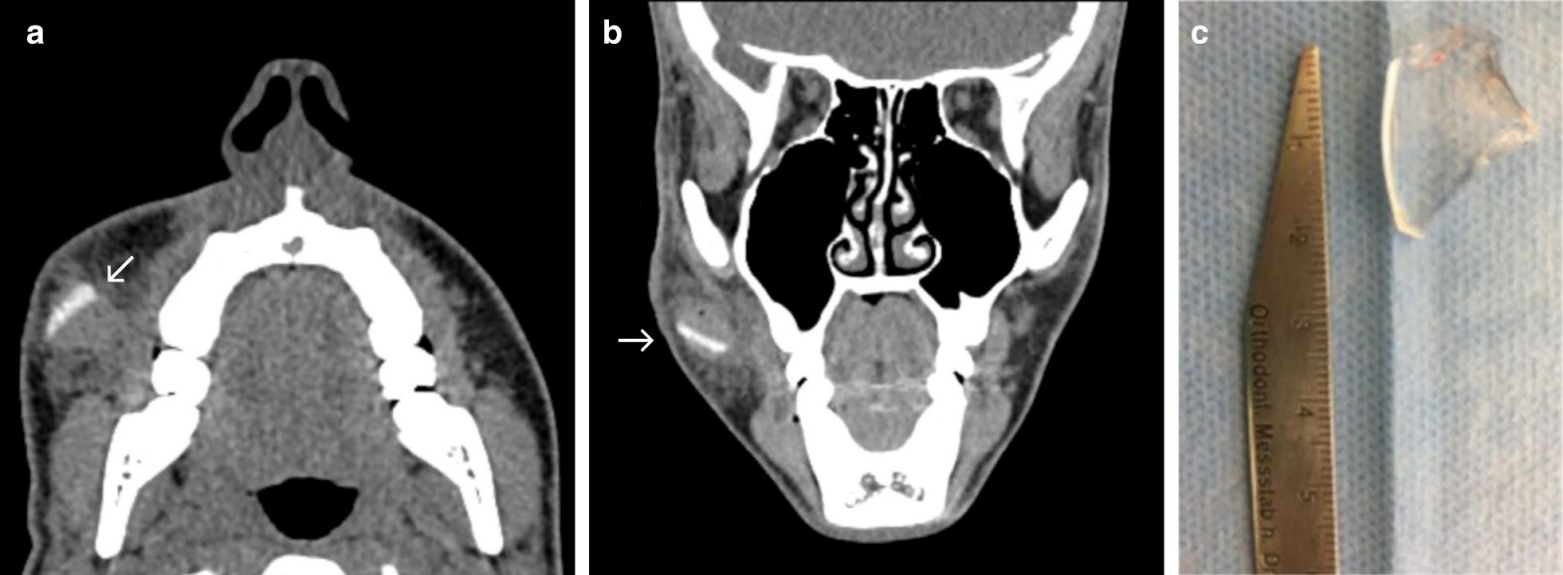
Broken ophthalmic lens. Axial (**a**) and coronal (**b**) CT images showing a hyperdense foreign object (arrow) in the right buccal area surrounded by haematoma. Photograph of a translucent piece of glass after surgical retrieval (**c**)

A 17-year old patient presented to our hospital with multiple injuries to his face after a bicycle accident. The patient reported that his glasses broke during the accident. Clinical examination revealed a 2 × 4 cm cut of his lower eyelid. A CT scan confirmed the existence of a dislocated hyperdense structure in his right buccal area (Fig. 11a, b). Deeper wound examination under local anaesthesia confirmed the presence of a dislocated, broken ophthalmic lens, which was removed under antibiotic treatment (Fig. 10c). The patient was discharged the day after without any complications.

### Case No. 12: Broken water glass

**Fig. 12:**
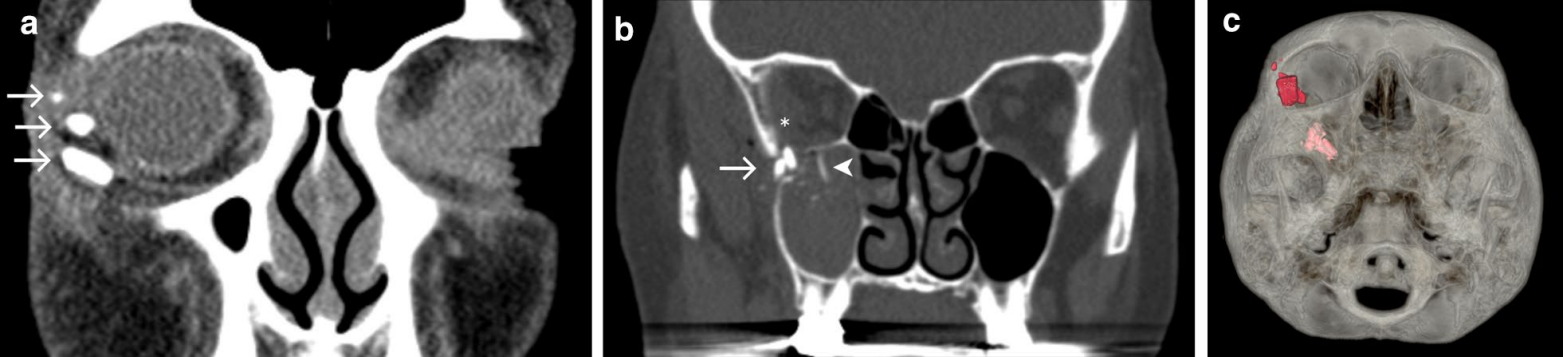
Broken water glass. Coronal CT sections (**a**, **b**) and 3-dimensional volume rendering (**c**) displaying intraorbital hyperdense foreign objects (arrows) in close proximity to the globe and the inferior oblique muscle (**a**) and inside the infratemporal fossa, protruding into the maxillary sinus and orbital cavity (**b**). Pronounced hyperdensity of the glass objects enables their differentiation from dislocated bone fragments (arrowhead). Also note intraorbital-subperiosteal haematoma (asterisk)

A 76-year-old female patient arrived at the emergency room after tumbling while holding a glass in her hand. Clinically, the upper right eyelid was ruptured. To rule out any foreign bodies as well as orbital injuries, a CT scan of the midface was performed. Besides multiple hyperdense fragments in intraorbital and periorbital locations (max. size 18 mm), fractures of the orbital floor were detected (Fig. 12). Multiple glass fragments were removed under general anaesthesia, and the lateral canthus was re-attached.

#### Plastic

Plastics are synthetic or semi-synthetic organic compounds that are based on high molecular mass polymers or prepolymers. Properties of polymers can be modulated by varying their molecular architecture or formulation. The term “plastic” therefore covers a wide variety of materials with different characteristics which need to be considered when comparing studies analysing the visibility of “plastic” objects in different set-ups.

Plastic foreign objects exhibit good visibility when displaying a hyperechoic signal during ultrasound scanning. Ingraham and colleagues reported difficulties in detection of plastic objects in MRI scans [31], while Pattamapaspong et al. found MRI to be the most reliable modality to detect plastic foreign objects in an experimental setting of the foot [35]. Javadrashid and colleagues analysed plastic objects with different sizes (0.5–3 mm) and concluded that CT imaging was the best modality for the detection of plastic foreign objects. They reported that conventional X-ray imaging, MRI and ultrasound scanning were only successful in detecting plastic that objects were larger than 2, respectively, 3 mm [32]. However, data about visibility are inconsistent and depend on material composition, sample size and study design [29, 31, 32, 35].

### Case No. 13: Plastic drainage tube

A 63-year-old male patient presented to our out-patient clinic with a swelling of the left of his neck close to an old scar. The patient’s history revealed an abscess treatment comprising the extraoral drainage of an abscess 30 years ago. A CT scan showed a foreign object within the patient alongside an acute abscess formation. Intraoperatively, plenty of pus was discharged and multiple parts of a retained plastic drainage tube were removed. Post-operatively, the patient was discharged without any complaints.

#### Stone

Depending on their mineral and chemical composition, stones bear different physical characteristics. Therefore, the imaging of various stones will have different imaging characteristics [61]. On radiographs and CT scans, stones are radiopaque objects with different densities [61]. Stone is best detected during CT imaging followed by conventional radiography [29, 32]. Even though stones normally demonstrate a radiodensity greater than that of bone, conspicuity might be reduced when the stone is in close proximity to bony structures [61]. Stone can also be detected in ultrasound scans when they result in a strong echo response, interface and acoustic shadow. In MRI scans, ferromagnetic mineral components can lead to artefacts [34].

Case 14 illustrates a retained stone in a patient who was hit by a car as pedestrian.

### Case No. 14: Stone/Gravel

**Fig. 13:**
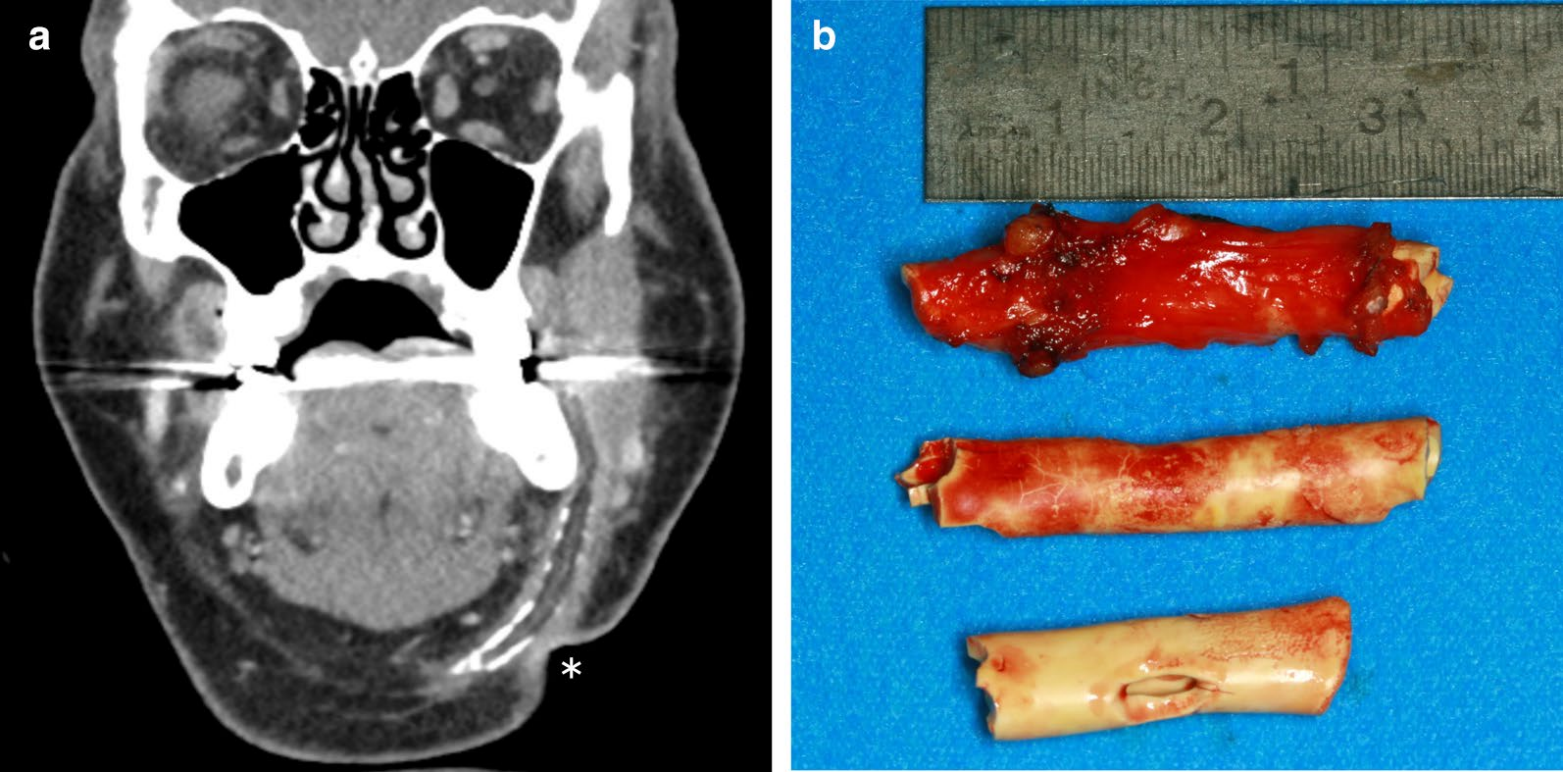
Plastic drainage tube. Coronal CT image (**a**) showing a tubular hypodense structure with appositional calcifications in the left submandibular space. Adjacent soft tissue reactions raise the suspicion of superinfection. Note the retraction of the soft tissue contour (asterisk), representing the old surgical scar. **b** Photograph of 3 parts of a drainage tube after removal

**Fig. 14:**
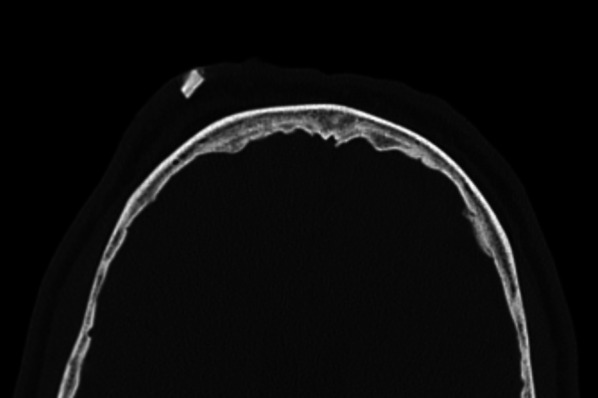
Stone. Axial CT showing a hyperdense structure in the subcutaneous tissues in the right frontal area

A 59-year-old patient was admitted to the emergency room after being hit by a car as a pedestrian. Clinically, the patient presented multiple facial injuries with soft tissue defects. Polytrauma CT scanning detected a hyperdense foreign object embedded in the frontal soft tissue (Figs. 13, 14). Under local anaesthesia, a 10 × 5 mm stone splinter was removed under antibiotic treatment.

#### Fish bones, scales and beaks

Fish bones exhibit a radiopaque signal in conventional X-ray and CT scans. However, depending on their anatomical position and size, their detection can be difficult. A non-treatment approach bears the risk of migration of foreign materials, and indeed, severe outcomes have previously been reported [18]. While X-ray imaging provides a good overview with little radiation involved, small fish bones in particular might be missed. CT scans allow a precise localisation of the anatomical position even of smaller objects, at the cost of higher radiation doses. Ultrasound scanning might be a good option, resulting in a hyperechoic signal [34]. One option could also be to select the imaging modality depending on the age of the patient [59]. Fish beaks and bills of various species can break during fish attacks bearing the risk of leading to dislocated foreign bodies with severe injury patterns [62, 63]. While scales and beaks might not be visible or could be misinterpreted in conventional X-ray scanning, CT imaging, with its optional 3D reconstructions might offer a better means of identifying foreign objects as well as localising their surrounding structures [2, 20]. Based on the bacterial flora of fishes, unnoticed foreign bodies injuries could lead to impaired wound healing and wound infections, therefore needing to be removed while undergoing antibiotic treatment [64].

### Case No. 15: Beak of a needlefish

**Fig. 15:**
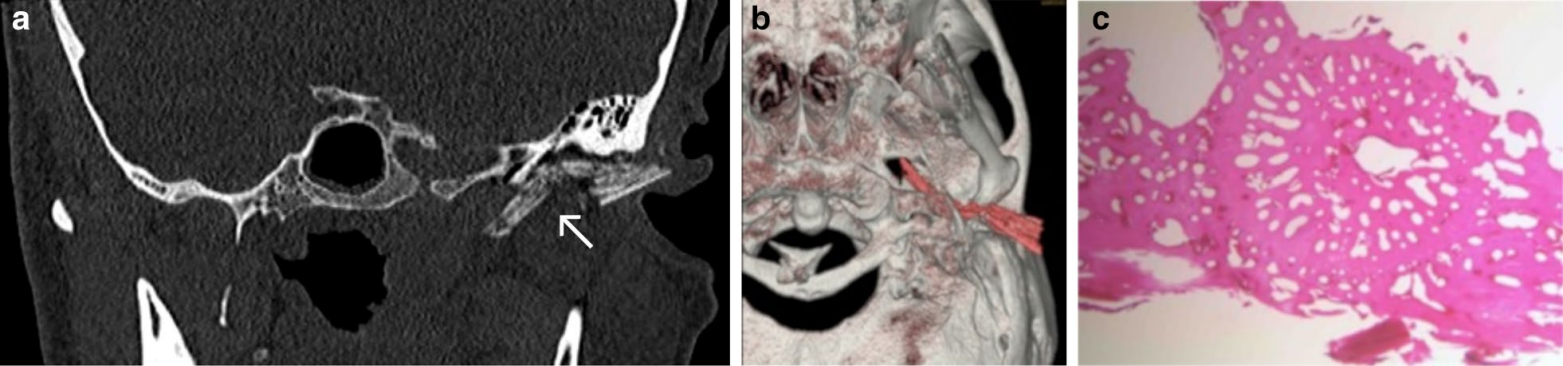
Beak of a needlefish. Coronal oblique CT image (**a**) and 3-dimensional volume rendering (**b**) showing a dislocated hyperdense foreign body (arrow) piercing through the auricle and traversing through the soft tissues rostral to the external acoustic meatus/posterior to the temporomandibular joint discus in close proximity to the petrotympanic fissure. The tip is visualised posterior to the lateral pterygoid muscle, implying its close relationship to the mandibular nerve and the maxillary artery. Photograph of histological analysis (HE staining) of stained foreign object (**c**)

A 46-year-old patient was admitted to our emergency department complaining about otalgia. Clinically, a fistula was detected below the antihelix and a defect of the left external ear canal. Purulent discharge provided evidence of a bacterial infection. Intravenous therapy was initiated, but the patient left the facility against medical advice. More than a month later, the patient presented to the emergency department with persisting symptoms. This time the patient provided a detailed history of an injury to his external auditory canal after being injured by a crocodile needlefish in Thailand. Accordingly, a CT scan of the head revealed the presence of a dislocated hyperdense foreign body (Fig. 15a, b). Histological analysis confirmed parts of the beak of a fish (Fig. 15c).

### Case No. 16: Fish scales

**Fig. 16:**
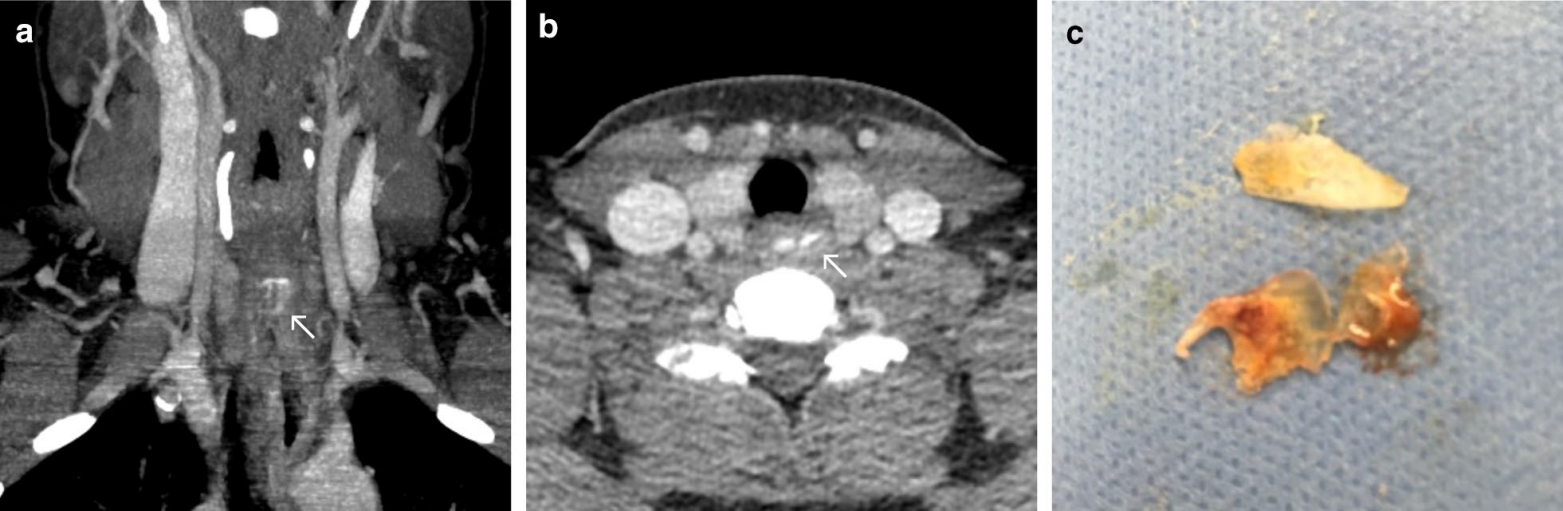
Fish scales. Coronal maximum intensity projection (**a**) and axial CT image (**b**) showing a flat hyperdense structure in the upper oesophageal sphincter. Photograph of multiple fish scales after endoscopic removal from the proximal oesophagus (**c**)

A 38-year-old female patient was transferred to our emergency department with dysphagia and odynophagia after eating a fish. Complaints persisted over days even though a soft diet was initiated. A clinical examination was insufficient due to the non-compliance of the patient. CT scanning presented hyperdense structures in the cervical oesophagus (Fig. 16a, b). Under general anaesthesia, 3 fish scales were removed from the oesophagus entrance (Fig. 16c). The patient was discharged without further complaints.

## What the clinician wants to know

- presence or absence of foreign bodies
- presumed material composition
- precise anatomic location
- complications: airway compromise, haematoma/active bleeding, vascular injury (dissection, pseudo-aneurysm, occlusion), organ injuries, emphysema, abscess
- concomitant injuries

## Conclusions

Clinical examination and diagnostic imaging are both crucial for the detection of foreign bodies in injuries of the head and neck areas. Different radiological modalities have strengths and limitations based on their underlying physical principles. However, various foreign objects including wood, plastics and glass might present diagnostic pitfalls, complicating their detection. In severe injuries with expected facial fractures, CT should be the modality of first choice. X-ray imaging can provide a low-radiation alternative to CT when the presumed foreign object is known to be radiopaque (e.g. metal, glass or stone), whereas ultrasound may be considered in superficial injuries. MRI should be used in inconclusive situations including the possible presence of a non-radiopaque object, persisting wound healing disorders or the involvement of the orbital cavity after excluding ferromagnetic foreign bodies.

## Data Availability

Data sharing is not applicable to this article as no datasets were generated or analysed during the current study.

## Abbreviations

3D: 3-Dimensional
BIH: Berlin Institute of Health
CT: Computed tomography
MRI: Magnetic resonance imaging
